# Oral to nasal endotracheal tube exchange using tracheal tube guide and video laryngoscope in a pediatric patient with facial burns: a case report

**DOI:** 10.1186/s12245-022-00451-3

**Published:** 2022-09-05

**Authors:** Naoki Yogo, Taeko Sasaki, Masato Kozumi, Yuya Kinoshita, Yuichiro Muto, Katsuki Hirai, Yuichiro Yoshino

**Affiliations:** 1 Department of Pediatrics, Japanese Red Cross Kumamoto Hospital, Kumamoto, Japan; 2 Division of Pediatric Emergency and Critical Care, Department of Pediatrics, Japanese Red Cross Kumamoto Hospital, 2-1-1 Nagamineminami, Higashi-ku, Kumamoto, 861-8520 Japan; 3 Department of Trauma Surgery, Japanese Red Cross Kumamoto Hospital, Kumamoto, Japan; 4 Department of Dermatology, Japanese Red Cross Kumamoto Hospital, Kumamoto, Japan

**Keywords:** Safe conversion, Difficult airway, Intubation, Tube exchanger

## Abstract

**Background:**

Airway management in children with severe burns is difficult because of airway edema and prolonged duration of ventilatory management. There is insufficient evidence to suggest that tracheostomy is beneficial for children.

**Case presentation:**

A male child aged 1 year and 4 months was injured when he accidentally fell into a bathtub filled with boiling water. Furthermore, 85% of the burnt area, including the face and neck, consisted of second-degree burns; hence, oral tracheal intubation and resuscitative infusion were required. In this case, the patient was safely switched from oral to nasotracheal intubation using a tracheal tube guide and video laryngoscope, without the use of a bronchoscope, and ventilatory management could be continued for 2 weeks.

**Conclusion:**

Oral to nasal endotracheal tube exchange using a tracheal tube guide and video laryngoscope may be useful not only for pediatric burn patients but also for adult patients who need to be safely switched from oral to nasotracheal intubation.

## Background

Replacing an oral tracheal tube with a nasal tracheal tube may be necessary during otolaryngological or dental surgery procedures. Postoperative pediatric cardiac patients often undergo nasotracheal intubation in the pediatric intensive care unit (PICU). According to a previous report, unplanned extubation occurred less frequently with nasal intubation than with oral intubation [1]. Furthermore, in the case of facial burns, it is not possible to fix the tube with tape, as is usually done, so it is necessary find creative ways to fix the tube [2–4]. Herein, we report a method of switching from oral to nasal intubation using a tracheal tube and video guide in a pediatric patient with acute facial burns.

## Case presentation

**Fig. 1:**
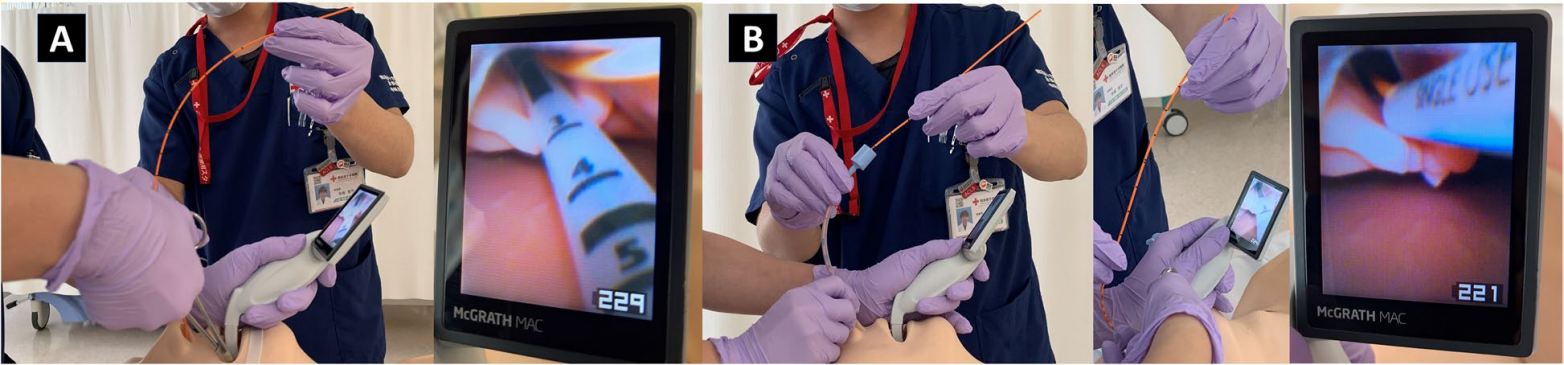
Oral to nasal endotracheal tube exchange. **A** A tube guide (Portex® tracheal tube guide 5CH) was inserted through the nose and grasped with Magill forceps while sharing the image of the glottis with the carer on McGrath™. The tube guide was passed adjacent to the oral tracheal tube. **B** The tracheal tube was passed through the tube guide and advanced close to the glottis. The oral tracheal tube was removed, and the nasotracheal tube was passed along the tube guide to the glottis

**Fig. 2:**
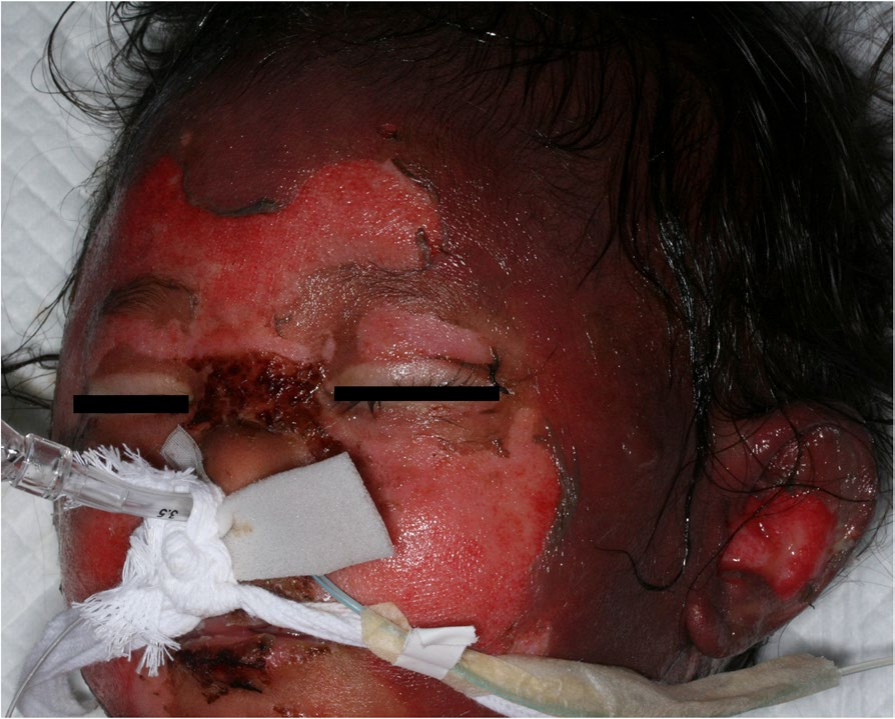
Fixation of tracheal tube. A string was threaded through the nasal wings and tied to the tracheal tube. A thick cord was passed around the back of the head, knotted in the middle of the upper lip, and tied to the tracheal tube

A male child aged 1 year and 4 months with no relevant medical history accidentally fell into a bathtub filled with boiling water at a lodging facility. Burns occurred in areas other than those covered by a diaper, 85% of which were second-degree burns. On arrival at the hospital, oral tracheal intubation was performed, and infusion therapy was initiated. The infusion rate was based on the Advanced Burn Life Support program and was adjusted according to urine output. Owing to the presence of facial burns, the tracheal tube could not be fixed with tape as usual, and replacement with a nasal intubation tube was required. The tracheal tube was replaced using a video laryngoscope (McGrath™). First, a nasally inserted tracheal tube guide (Portex® tracheal tube guide 5CH) was advanced into the trachea using Magill forceps and placed adjacent to the oral tracheal tube. Next, the tracheal tube was advanced from the nasal cavity to the glottis via the tracheal tube guide. Finally, the original tracheal tube was removed, and the nasal tracheal tube was advanced into the trachea and fixed in place (Fig. 1). The replacement tracheal tube was secured with a cord over the upper lip and a thread over the nasal wing (Fig. 2). On the 4th hospital day, his systolic blood pressure decreased to 60 mmHg, and pancytopenia was observed. We concluded that the patient was in septic shock; hence, he was started on meropenem and vancomycin, and his circulation was stabilized with noradrenaline. As the IgG level decreased to 87 mg/dL, intravenous immunoglobulin at 0.5 g/kg was administered on day 3. On the 7th day, noradrenaline was discontinued, and debridement was performed. On the 14th day, he was extubated, and on the 21st day, the wounds were re-covered with artificial dermis after debridement. An autologous epidermis culture was implanted on the 28th day, and he was discharged on the 41st day.

## Discussion

Our patient was safely switched from oral to nasal intubation using a tracheal tube guide. Although nasotracheal intubation was a suitable option, there were concerns regarding upper airway edema due to the large burn area and facial burns. Since the patient was in the acute phase of a burn injury, a tracheal tube guide was used considering the risk of difficult airway management. A tracheal tube can be safely managed for 2 weeks without tracheostomy in pediatric patients with facial burns.

In children, nasal intubation is not only used in otolaryngological and dental surgery but also used in the PICU for post-cardiac surgery management, since unplanned extubation is reported to be less frequent with nasal intubation than with oral intubation [1]. Nasotracheal intubation is generally associated with less stimulation of pharyngeal reflex and less discomfort than endotracheal intubation; in addition, with the former, oral care is easier, and the tubes are less likely to bend and are less likely to be bitten. With endotracheal intubation, it is difficult to secure the tracheal tube, and there is a risk of accidental extubation due to tongue movement. Hence, nasotracheal intubation is beneficial for children in whom it is difficult to maintain the tracheal tube position [5].

In burn patients, tracheal intubation should be considered in cases of airway burns, burn area of more than 40%, or burns of the face or neck [6, 7]. In children, fatality rates have been reported to increase when the burn area exceeds 60% [8]. In adults, tracheostomy should be considered for patients with severe burns, as in the present case, because prolonged ventilatory management is expected. However, few studies have shown the benefits of tracheostomy in children [9]. Regardless of the indications, tracheostomy is not recommended for children because it can lead to delayed language development [10]. In addition, if there are burns on the anterior neck, tracheotomy should be performed after skin grafting [11]; hence, airway management techniques without tracheostomy are needed for pediatric patients with severe burns, including facial and cervical burns.

Various methods for switching from oral to nasal intubation have been reported [12–15]. Many methods involve bronchoscopy [12, 15] or a short period of airway loss. Children are more prone to obstruction by airway edema than adults; therefore, an appropriate method of tracheal tube replacement is required for such pediatric patients [5]. The method adopted in our patient’s case can be performed using a McGrath™ laryngoscope, which allows images to be shared with the carers; unlike bronchoscopy, tracheal tube replacement can be performed in a limited workspace because the McGrath™ is small and does not require a separate monitor. In addition, by using a tracheal tube guide, a stable airway can be maintained until just before replacement, with virtually no loss of airway security.

Various tube fixation methods for facial burns have been reported [2–4]. In the current case, nasotracheal intubation was considered less likely to cause unplanned extubation than oral intubation; however, the method of fixation required ingenuity. As the tape could not be fixed to the skin due to the burns, a string was used for fixation, and a thread was applied to the nasal wings to prevent the tracheal tube from moving.

The child with severe burn injury was safely switched from oral to nasotracheal intubation, and ventilatory management was continued for 2 weeks, despite burns to the face and neck. Safe tracheal tube replacement is possible with a tracheal tube guide and video laryngoscope without the use of a bronchoscope. This method may allow a safe transition from oral intubation in patients requiring nasal intubation, especially pediatric patients with facial burns, and help prevent unexpected extubation.

## Data Availability

Not applicable.

## Abbreviations

DAM: Difficult airway management
ABSL: Advanced Burn Life Support
PICU: Pediatric intensive care unit
